# Salmon Fillet Intake Led to Higher Serum Triacylglycerol in Obese Zucker Fa/Fa Rats But Not in Normolipidemic Long-Evans Rats

**DOI:** 10.3390/nu10101459

**Published:** 2018-10-08

**Authors:** Linn Anja Vikøren, Aslaug Drotningsvik, Svein Are Mjøs, Gunnar Mellgren, Oddrun Anita Gudbrandsen

**Affiliations:** 1 Dietary Protein Research Group, Department of Clinical Medicine, University of Bergen, 5020 Bergen, Norway; linn.anja.slake.vikoren@helse-bergen.no (L.A.V.); aslaug.drotningsvik@uib.no (A.D.); 2 Department of Clinical Science, University of Bergen, Haukeland University Hospital, 5020 Bergen, Norway; 3 Department of Chemistry, University of Bergen, P.O. Box 7803, 5020 Bergen, Norway; svein.mjos@uib.no; 4 Nofima BioLab, P.O. Box 1425 Oasen, 5828 Bergen, Norway; 5 Department of Clinical Science, KG Jebsen Center for Diabetes Research, University of Bergen, Haukeland University Hospital, 5021 Bergen, Norway; gunnar.mellgren@uib.no; 6 Hormone Laboratory, Haukeland University Hospital, 5021 Bergen, Norway

**Keywords:** n-3 PUFA, salmon, fatty fish, apoB48, apoB100, triacylglycerols, phospholipids, fatty acids, Zucker fa/fa rat, Long-Evans rat

## Abstract

The triacylglycerol lowering effect of fatty fish and fish oils is well recognized, however we recently showed that salmon intake resulted in higher serum triacylglycerol concentration in obese Zucker fa/fa rats. Since effects of salmon fillet have never before been studied in rats, the objective of this study was to compare effects of salmon intake on serum lipids in hyperlipidemic obese rats with normolipidemic lean rats. Zucker fa/fa rats and Long-Evans rats were fed diets with 25% protein from baked salmon fillet and 75% protein from casein, or casein as sole protein source (control group) for four weeks. Serum triacylglycerol concentration was higher, and cholesterol and apolipoproteinB-100 concentrations were lower in Zucker fa/fa rats fed Baked Salmon Diet compared to Zucker fa/fa rats fed Control Diet, with no differences in serum triacylglycerol, cholesterol and apolipoproteinB-100 between Long-Evans rats fed Baked Salmon Diet or Control Diet. Serum triacylglycerol fatty acid composition showed greater similarities to dietary fatty acids in Zucker fa/fa rats than in Long-Evans rats. To conclude, intake of baked salmon fillet resulted in higher serum triacylglycerol concentration and lower serum cholesterol concentration in hyperlipidemic obese Zucker fa/fa rats but did not affect serum lipids in normolipidemic lean Long-Evans rats.

## 1. Introduction

Elevated concentrations of fasting and non-fasting triacylglycerols are independent risk factors of cardiovascular disease [1]. Intake of long chain n-3 PUFAs has been found to have cardio protective effects, especially in patients with dyslipidemia [2]. The reduction in circulating triacylglycerol is considered the most consistent effect of n-3 PUFA consumption [3,4,5,6,7,8,9,10,11,12,13,14,15,16,17,18], but there is controversy as to the cholesterol-regulating effects of the marine n-3 fatty acids [9,11,13,14,15,16,19,20,21]. Several types of hyperlipidemia were defined by Fredrickson and Lees [22], with elevated concentrations of various types of lipids and lipoproteins. In the context of the present paper, Types I and V hyperlipidemia are of special interest. Type I hyperlipidemia is characterized by severely elevated levels of chylomicrons, and thus high circulating levels of cholesterol and especially triacylglycerols, and is caused by mutations of either the gene for lipoprotein lipase or its cofactor apolipoprotein C-II [22]. In patients with Type V hyperlipidemia, concentrations of both chylomicrons and VLDL are elevated, accompanied with elevated serum levels of cholesterol and both endogenous and exogenous triacylglycerols, with normal or modestly reduced lipoprotein lipase activity [22].

The obese Zucker fa/fa rat is a much used animal model for studies of metabolic complications and possible treatments of obesity, since it has an abnormal lipid metabolism and presents changes often seen in human obesity [23]. Visible obesity is present already 3–5 weeks after birth, and in addition the obese Zucker rats develop a range of endocrine abnormalities resembling human metabolic syndrome, including insulin resistance, dyslipidemia, mild glucose intolerance and hyperinsulinemia [23]. The obese Zucker fa/fa rat may be an interesting model for hyperlipidemias of Type I and V, as it has an elevated secretion of triacylglycerols in VLDL from liver combined with a slow clearance of chylomicrons, resulting in high circulating concentrations of VLDL and chylomicrons, and consequently high levels of lipids including triacylglycerols and cholesterol compared to metabolic healthy rats [24,25].

We have recently shown that, when obese Zucker fa/fa rats were fed a diet containing 25% of the protein from baked salmon fillet, it resulted in a significantly higher serum triacylglycerol concentration when compared to a standard control diet with casein as the sole protein source [26]. To our best knowledge, this is the first study on salmon fillet fed to two different rat strains. Since n-3 PUFA consumption has been repeatedly shown to reduce circulating triacylglycerol concentration [3,4,5,6,7,8,9,10,11,12,13,14,15,16,17,18], the objective of the present study was to compare the effects of intake of baked salmon on serum lipids in hyperlipidemic obese Zucker fa/fa rats with that of lean Long-Evans rats which have normal lipid metabolism. Our hypothesis was that the high serum triacylglycerol concentration in obese Zucker fa/fa rats fed salmon diet was a consequence of the slow clearance of chylomicrons in these rats [24,25] and thus the fatty acid composition in serum triacylglycerols would be a reflection of the dietary fatty acids. Thus, we did not expect differences in serum lipids and apoBs concentrations between Long-Evans rats fed baked salmon fillet and control diet since these rats have normal lipid metabolism.

## 2. Materials and Methods

### 2.1. Ethical Statement

The study protocol was approved by the National Animal Research Authority of Norway, in accordance with the Animal Welfare Act and the regulation of animal experiments (approval no 2014–6979). The animal care and use program at the Faculty of Medicine at University of Bergen is accredited by the Association for Assessment and Accreditation of Laboratory Animal Care International.

### 2.2. Design

Two experiments were conducted. In the first experiment, twelve male Zucker fa/fa rats (HsdHlr:ZUCKER-Leprfa, from Harlan Laboratories, Indianapolis, IN, USA) were assigned to two experimental groups of six rats each with comparable mean bodyweight. Some data from this experiment, including serum lipids, have already been published in Vikøren et al. [26] and Vikøren et al. [27], and the finding that a Baked Salmon Diet resulted in a significantly higher serum triacylglycerol concentration when compared to a standard Control Diet in obese Zucker fa/fa rats was the background for comparing these diets in another rat model. Therefore, in a second experiment, twelve male Long-Evans rats (Crl:LE, from Charles River Laboratories, Calco, Italy) were assigned to two experimental groups of six rats each with comparable mean bodyweight. The rats in the two experiments were treated in the exact same manner and therefore the designs of the two experiments are presented together. Rats were housed in pairs in a room maintained at a 12 h light–dark cycle (light from 07:00 to 19:00) with constant temperature of 20 ± 3 °C and relative humidity of 65 ± 15%. The rats were acclimatized for at least one week under these conditions before the start of the experiments.

The intervention period in the Zucker fa/fa rat study started when the rats weighed 350 ± 20 g (i.e., 8–9 weeks old), and after the four-week intervention period the Zucker fa/fa rats weighed 554 ± 26 g. To make certain that differences in serum lipids between the strains were not a consequence of differences in bodyweights, we wanted the Long-Evans rats to reach a similar bodyweight as the Zucker rats after four-week intervention, and based on the growth chart from Charles River Laboratories the starting weight of the Long-Evans rats should be around 440 g. The intervention in the Long-Evans rat study started when the rats weighed 430 ± 30 g (i.e., when the rats were 13–15 weeks old) and the mean body weight for all Long-Evans rats at endpoint was 572 ± 38 g.

Rats were weighed weekly during the intervention period. One week before euthanasia, rats were housed individually in cages with grids for 24 h without fasting in advance for measurements of feed intake.

### 2.3. Diets

The rats were fed modified, semi-purified experimental diets prepared according to the instructions for the AIN-93G [28] with addition of 1.6 g methionine/kg diet as recommended by Reeves [29] (Table 1) for four weeks. The Control Diet contained 20 wt % proteins from casein (Sigma-Aldrich, Munich, Germany). Skin free salmon fillets from Atlantic Salmon (farmed Salmo salar, fed 6% EPA+DHA of fatty acids until 1200 g bodyweight and 4.5% EPA+DHA in following feeds [30]) were provided by Leroy Seafood Group (Hordaland, Norway). Baked salmon filets were prepared in oven at 180 °C for 20 min (no oil or fat were added when baking the fish) and were minced, freeze dried and grinded before it was mixed with the other ingredients in the diets. All other ingredients were purchased from Dyets Inc. (Bethlehem, PA, USA). All rats had free access to tap water and feed (ad libitum). The feed was contained in ceramic bowls that were too heavy for the rats to knock over. Feed leftovers were thrown away and newly thawed feed were provided every day. Since the feed was given as a powder formula, the rats had access to wood chewing sticks.

### 2.4. Euthanasia and Sampling

The rats were euthanized while under anesthesia with isoflurane (Isoba vet, Intervet, Schering-Plough Animal Health, Boxmeer, The Netherlands) mixed with nitrous oxide and oxygen after four-week intervention, after a 12-h fast with free access to tap water. Blood was drawn directly from the heart and collected in Vacuette Z Serum Clot Activator Tubes (Greiner Bio-one) for isolation of serum. Epididymal, renal and retroperitoneal white adipose tissues (WAT) were dissected out and weighed. Serum and WAT were snap-frozen in liquid nitrogen and stored at −80 °C until analysis.

### 2.5. Analyses of Diets

The dietary contents of amino acids, total fat (Folch extraction) and total energy in the diets were analyzed by Nofima BioLab (Bergen, Norway), and the fatty acid composition in the diets were analyzed by gas chromatography as previously described [31,32,33,34,35], and have been published earlier [26]. In brief, the dietary contents of indispensable amino acids were in general similar in Control Diet and Baked Salmon Diet, whereas taurine was found only in the Baked Salmon Diet. The Baked Salmon Diet contained higher amounts of 16:0, 18:0, 18:1n−7, 18:1n−9, 18:2n−6 and 18:3n−3 when compared to the Control Diet, and the long chain n−3 PUFAs 20:5n−3, 22:5n−3 and 22:6n−3 were found only in the Baked Salmon Diet (Table 2).

### 2.6. Analyses in Serum

Fasting serum concentrations of triacylglycerols, total cholesterol, LDL cholesterol, HDL cholesterol, alanine transaminase and total bile acids were analyzed by accredited methods at the Laboratory of Clinical Biochemistry at Haukeland University Hospital (Bergen, Norway) on the Cobas 8000 c702 module from Roche Diagnostics GmbH, Mannheim, Germany using the TRIGL, CHOL2, LDL_C, HDLC3 and ALTPM kits from Roche Diagnostics GmbH and the Total bile acids assay kit from Diazyme Laboratories, Inc., Poway, CA, USA. Non-esterified fatty acids, free cholesterol and aspartate transaminase were analyzed on the Cobas c 111 system (Roche Diagnostics GmbH, Mannheim, Germany) using the NEFA FS kit (DiaSys, Diagnostic Systems GmbH, Holzheim, Germany), the free cholesterol FS kit (DiaSys) and the Aspartate aminotransferase ASTL kit (Roche Diagnostics GmbH). Serum cholesteryl ester was calculated as the difference between total and free cholesterol. Apolipoprotein B48 and B100 were measured using the MBS753664 Rat Apolipoprotein B48 ELISA Kit (MyBioSource Inc., San Diego, CA, USA) and the MBS723231 Rat Apoprotein B100 ELISA Kit (MyBioSource). C-reactive protein (CRP) was measured with the Rat C-Reactive Protein ELISA kit (#88-7501, from Invitrogen by Thermo Fisher Scientific Inc., Carlsbad, CA, USA). Serum insulin was measured with the Insulin (Rat) Elisa kit (EIA-2048, from DRG Instruments GmbH, Marburg, Germany). Serum concentrations of triacylglycerol, total cholesterol, LDL cholesterol, HDL cholesterol, total bile acids, NEFA and cholesteryl ester in these Zucker fa/fa rats are previously published [26].

### 2.7. Analyses of Fatty Acids in Serum Lipids

Lipids were extracted from serum by the method of Bligh and Dyer [31] using a mixture of chloroform and methanol, and lipid classes were separated by TLC on silica gel plates (250 µm Silica gel 60 from Merck KGaA, Darmstadt, Germany) developed in hexane–diethyl ether–acetic acid (40:10:1, by vol) [36]. The triacylglycerols and phospholipids spots were identified using Rhodamine G (Fluka Chemie AG, Buchs, Switzerland) and co-migration with known standards, and were scraped off, methylated and analyzed as described previously [32,33,34,35].

### 2.8. Statistical Analysis

Statistical analyses were conducted using SPSS Statistics version 25 (SPSS, Inc., IBM Company, Armonk, NY, USA). Groups within rat strains were compared using Independent Samples T Test assuming equal variances. One-way ANOVA was used to compare bodyweight at baseline, bodyweight at endpoint and energy intake for all groups (strain and diets), with Tukey HSD post hoc test when appropriate. The cut-off level for statistical significance was taken at a probability of 0.05. Rats fed a casein-based diet served as controls in both experiments. One Zucker fa/fa rat in the Control Group in the first experiment had to be euthanized due to a serious lesion and poor health in the first week of the study and is not included in the results, therefore *n* = 5 in the Control Group and *n* = 6 in the Baked Salmon Group. In the second experiment (Long-Evans rats), *n* = 6 in both Baked Salmon Group and Control Group. Data are presented as mean ± standard deviation.

## 3. Results

### 3.1. Bodyweight, Growth, White Adipose Tissue Weight, and Energy and Fat Intake

**Figure 1:**
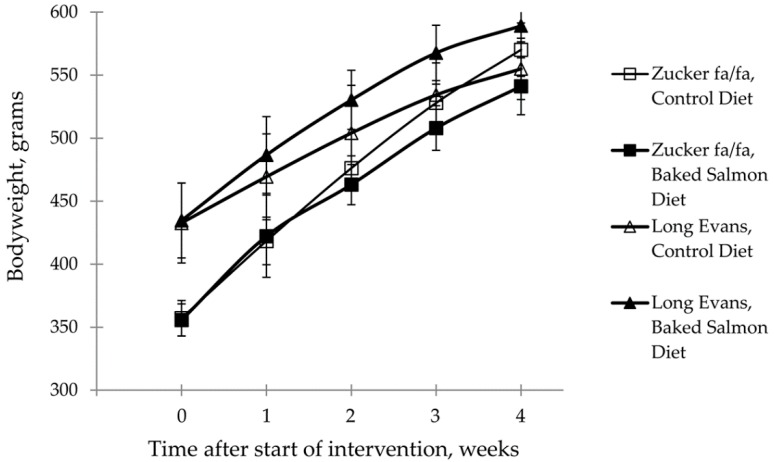
Bodyweight development (grams) in obese Zucker fa/fa rats and Long-Evans rats, shown as mean values with standard deviations, for *n* = 5 in Zucker fa/fa Control Group, and *n* = 6 in all other groups. No differences were seen at any time points within any of the rat strains. *p* < 0.05 was considered significant. Groups within rat strains are compared using Independent Samples T Test assuming equal variances.

The mean bodyweight at baseline was similar between Zucker fa/fa rats in Baked Salmon Group and in Control Group (*p* = 0.85), and was also similar between Long-Evans rats in Baked Salmon Group and in Control Group (*p* = 0.92, Figure 1). The study was designed so that the Zucker fa/fa rats and the Long-Evans rats would reach the same bodyweight after four-week intervention, based on the growth charts from the breeders showing a much steeper growth curve for obese Zucker fa/fa rats, and therefore the bodyweight at baseline was significantly higher in the Long-Evans rats (*p* ANOVA = 2.2 × 10^−5^). At endpoint (four weeks), the mean bodyweight was similar in all groups (*p* ANOVA = 0.062). The mean bodyweights were similar between Zucker fa/fa rats fed Control Diet and Baked Salmon Diet, and between Long-Evans rat fed Control Diet and Baked Salmon Diet after one, two, three and four weeks (Figure 1).

The relative WAT-to-bodyweight ratio was similar in Zucker fa/fa rats fed Baked Salmon Diet or Control Diet, and was also similar in Long-Evans rats fed Baked Salmon Diet or Control Diet, but Zucker fa/fa rats had significantly more WAT compared to Long-Evans rats, independent of diets (*p* ANOVA = 8.9 × 10^−4^) (Table 3). The mean daily energy intake was similar in all groups (*p* ANOVA = 0.24), however the daily fat intake was significantly higher in both Zucker fa/fa rats and Long-Evans rats fed Baked Salmon Diet when compared to their respective control group.

### 3.2. Serum Analyses

**Figure 2:**
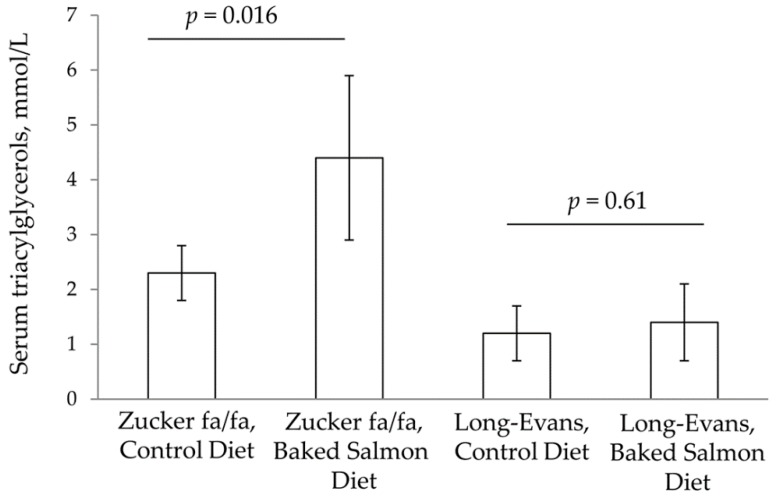
Serum triacylglycerols in obese Zucker fa/fa rats and Long-Evans rats, shown as mean values with standard deviations, for *n* = 5 in Zucker fa/fa Control Group, and *n* = 6 in all other groups. *p* values are shown for comparisons within rat strains. *p* < 0.05 was considered significant. Groups within rat strains are compared using Independent Samples T Test assuming equal variances.

In the Zucker fa/fa rat experiment, rats fed Baked Salmon Diet had significantly higher serum concentration of triacylglycerols, as we have previously published [26], whereas no differences were seen in serum concentrations of triacylglycerols between the Control Group and the Baked Salmon Group in the Long-Evans rat experiment (Figure 2). Zucker fa/fa rats fed Baked Salmon Diet had significantly lower serum concentrations of total cholesterol, HDL cholesterol, LDL cholesterol, cholesteryl ester and total bile acids when compared to those fed Control Diet, with no difference between the groups for serum NEFA concentration (Table 4), as we have previously published [26]. In contrast, in the Long-Evans rat experiment no differences were seen in serum concentrations of total cholesterol, HDL cholesterol, LDL cholesterol, cholesteryl ester and total bile acids between rats fed the Baked Salmon Diet or the Control Diet, however serum NEFA concentration was significantly lower in the Baked Salmon Group. Serum concentrations of triacylglycerols, total cholesterol, HDL cholesterol, LDL cholesterol, cholesteryl ester and total bile acids were significantly higher in Zucker fa/fa rats fed Control Diet when compared to Long-Evans rats fed the same diet (all *p* values < 1.0 × 10^−3^).

Serum apoB-48 concentrations were not different between Zucker fa/fa rats fed Baked Salmon Diet or Control Diet, whereas the serum concentration of ApoB-100 was significantly lower in Zucker fa/fa rats fed Baked Salmon Diet when compared to those fed Control Diet (Table 4). ApoB-48 and apoB-100 serum concentrations were similar between Long-Evans rats fed Control Diet or Baked Salmon Diet. Fasting insulin was measured only in Zucker fa/fa rats, and showed no difference between the dietary groups (Control Group 9.4 ± 7.1 μg/L; Baked Salmon Group 4.8 ± 2.3 μg/L, *p* = 0.16).

In Long-Evans rats, no differences were seen between the dietary groups for serum concentrations of alanine transaminase, aspartate transaminase (*p* values of 0.79 and 0.97, respectively, data not presented) and CRP (Table 4), whereas serum concentration CRP was significantly lower in Zucker fa/fa rats fed Baked Salmon Diet when compared to Zucker fa/fa rats fed Control Diet (Table 4). Serum concentration of aspartate transaminase was significantly lower in Zucker fa/fa rats fed Baked Salmon Diet when compared to Zucker fa/fa rats fed Control Diet (*p* = 0.017), whereas no difference was seen between the Zucker fa/fa diet groups in regard to alanine transaminase concentration (*p* value 0.28), as we have previously published [27]. When serum concentrations of alanine transaminase, aspartate transaminase and CRP were compared between Zucker fa/fa rats and Long-Evans rats fed Control Diet, concentrations were significantly higher in Zucker fa/fa rats (all *p* values < 1.0 × 10^−3^).

### 3.3. Fatty Acids in Triacylglycerols and Phospholipids in Serum

**Figure 3:**
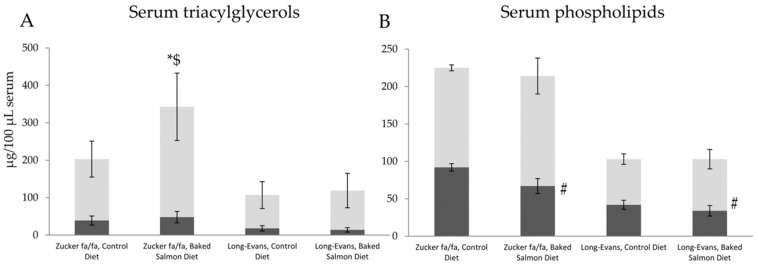
Fatty acids esterified as triacylglycerols (**A**) and phospholipids (**B**) in serum presenting fatty acids also found in diets (light grey bars) and fatty acids not found in diets (dark grey bars), shown as mean values with standard deviations, for *n* = 5 in Zucker fa/fa Control Group, and *n* = 6 in all other groups. *p* < 0.05 was considered significant. Groups within rat strains are compared using Independent Samples T Test assuming equal variances. * Total amount of fatty acids was significantly different from Control Group (same rat strain); $ amount of fatty acids found in the diet was significantly different from Control Group (same rat strain); and # amount of fatty acids not found in the diet was significantly different from Control Group (same rat strain).

As would be expected from the higher serum triacylglycerol concentration in Zucker fa/fa rats fed Baked Salmon Diet when compared to those fed Control Diet, the sum of fatty acids esterified as triacylglycerols were significantly higher in the Baked Salmon Group compared to the Control Group (343 ± 104 and 203 ± 59 µg/100 µL serum, respectively, *p* = 0.026), with no difference between these two groups for the total amount of fatty acids esterified as phospholipids (214 ± 32 and 225 ± 9 µg/100 µl serum, respectively, *p* = 0.49, Figure 3). In Long-Evans rats, no differences were seen in the sum of fatty acids esterified as triacylglycerols and phospholipids between Baked Salmon Group and Control Group (p values of 0.67 and 0.96, respectively).

In Zucker fa/fa rats, the higher amount of fatty acids as serum triacylglycerols in Baked Salmon Group when compared to Control Diet seems to originate from the diet (*p* = 0.018, Figure 3A), with no difference between these groups when comparing fatty acids esterified as triacylglycerols that were not identified in the diets (*p* = 0.26). In regard to fatty acids esterified as phospholipids in serum, no difference was seen between the Zucker fa/fa diet groups for fatty acids that were identified in the diets (*p* = 0.23, Figure 3B), however a significantly lower amount of fatty acids not found in the diets were esterified as phospholipids in serum of Zucker fa/fa rats fed Baked Salmon Diet (*p* = 6.7 × 10^−4^).

In Long-Evans rats, no difference was seen between the Control Diet and the Baked Salmon Diet for fatty acids esterified as triacylglycerols, and this applied both to those fatty acids that were found in the diets (*p* = 0.52) and those that were not found in the diets (*p* = 0.32). In addition, comparable amounts of fatty acids found in the diets were esterified as phospholipids in Long-Evans rats fed Baked Salmon Diet or Control Diet (*p* = 0.23), whereas a significantly lower amount of fatty acids not found in the diets were esterified as phospholipids in Long-Evans rats fed Baked Salmon Diet when compared to those fed the Control Diet (*p* = 0.039).

The relative amounts of individual fatty acids esterified as triacylglycerols and phospholipids are presented in Table S1. Of special interest in the current setting is the effect of salmon diet on long-chain n−3 PUFAs, showing that intake of Baked Salmon Diet led to significantly higher amounts of 20:5n−3, 22:6n−3 and total n−3 PUFAs as triacylglycerols and phospholipids in both rat strains. In addition, both Zucker fa/fa rats and Long-Evans rats fed Baked Salmon Diet had significantly higher amounts of 22:5n−3 in triacylglycerols, while Long-Evans rats but not Zucker fa/fa rats fed Baked Salmon diet had more 22:5n−3 in phospholipids when compared to their respective control groups.

## 4. Discussion

The present study is the first to compare the effects of a Baked Salmon Diet and a Control Diet in two very metabolically different rat models: the obese Zucker fa/fa rat which develops dyslipidemia and obesity at a young age [23,24] and the lean Long-Evans rat which has a normal lipid metabolism. Our findings show that the effects of salmon intake on lipid metabolism are very different in these two rat models even with similar bodyweight at end of study. The Zucker fa/fa rats fed the Baked Salmon Diet had higher serum concentrations of triacylglycerols and lower concentrations of total, HDL and LDL cholesterols, cholesteryl ester and total bile acids when compared to the Control Group, whereas no differences were seen between the two Long Evans groups fed Baked Salmon Diet or Control Diet in these parameters. The fatty acid composition in serum triacylglycerols reflected the dietary fatty acids to a larger degree in the obese Zucker fa/fa rats compared to the Long-Evans rats, and as expected, the contents of n−3 long-chain PUFA in serum triacylglycerols and phospholipids were higher in both rat strain groups fed the Baked Salmon Diet.

The reduction in circulating triacylglycerol is considered the most consistent effect of n-3 PUFA consumption, and beneficial effects of fish (especially fatty fish) and fish oil intake on lipid concentrations have been reported in both humans and animal models [3,4,5,6,7,8,9,10,11,12,13,14,15,16,17,18]. We have recently shown that, when these obese Zucker fa/fa rats were fed a diet containing baked salmon, it resulted in a higher serum triacylglycerol concentration when compared to a control diet containing casein and soy oil as the only sources of protein and fat [26]. This inspired us to repeat the experiment in Long-Evans rats which have a normal lipid metabolism, and to expand the analyses to include quantification of apolipoproteins and fatty acids in isolated serum triacylglycerols and phospholipids.

Zucker fa/fa rats are said to be hyperphagic [23], however in the present study the daily energy intake was similar to that of Long-Evans rats, with no differences between the dietary groups within each rat strain. Also, since the Baked Salmon Diet contained more fat than the Control Diet, the daily fat intake was higher in both rat strains fed salmon diet when compared to controls. Still, bodyweight was similar between all groups at endpoint, as anticipated, with higher relative amount of body fat in the Zucker fa/fa rats compared to Long-Evans rats and no apparent effect on adiposity after salmon intake was observed in either rat strains.

The obese Zucker fa/fa rat and the Long-Evans rat are two metabolically different rat strains, as was evident from the much higher adiposity and serum concentrations of lipids, CRP, alanine transaminase and aspartate transaminase observed in the Zucker fa/fa rats, in addition to the differences in serum triacylglycerol and phospholipid fatty acid compositions. It is well known that the high hepatic secretion of triacylglycerols and a low clearance of chylomicrons combined with low lipoprotein lipase activity in brown adipose tissue, skeletal muscle and cardiac muscle make the Zucker fa/fa rat hypertriglyceridemic at a young age. In contrast, the lipid metabolism in the Long-Evans rat is believed to be normal. Although Zucker fa/fa rats have high lipoprotein lipase activity in white adipose tissue, it is evident that the high lipoprotein lipase activity in WAT is not sufficient to compensate for the elevated hepatic triacylglycerol secretion [24,25]. The Zucker fa/fa rat is of great interest as a model of hyperlipidemia in humans, especially for type I and V which are characterized by elevated serum levels of chylomicrons (types I and V) and VLDL (type V), and low (type I) or normal/low (type V) lipoprotein lipase activity [22]. Dyslipidemia is associated with increased risk of developing CVD, and intake of long chain n−3 PUFAs has been shown to have cardio protective effects, especially in patients with dyslipidemia [2].

We were surprised to find that a diet containing baked salmon actually led to a higher serum triacylglycerol in these Zucker fa/fa rats when compared to a control diet [26]. Higher circulating triacylglycerol concentrations in Zucker fa/fa rats have also been reported after intake of diets containing either krill oil or fish oil, and it was suggested that this was caused by upregulation of VLDL-secretion from liver, stimulated by long-chain n−3 PUFAs [37]. However, others have shown that fish oil may actually reduce secretion of VLDL from the liver [38] and accelerate chylomicron triglyceride clearance [39], and reduction in circulating triacylglycerol is indeed considered the most consistent effect of n−3 PUFA consumption [16].

The effects of Baked Salmon Diet on serum lipids were very different between the two rat strains, with lower serum concentrations of cholesterols and bile acids when compared to the Control Diet in Zucker fa/fa rats, and no differences in serum lipid concentrations between Long-Evans rats fed the two diets. In fact, the only difference in serum lipids between Long-Evans rat fed Baked Salmon Diet and Control Diet was a lower serum NEFA concentration in the former. Several studies have shown that fish oil intake reduces circulating NEFA concentrations [38], which is of interest since elevated NEFA concentration is suggested to be a risk factor for cardiovascular disease [40].

The higher serum triacylglycerol in Zucker fa/fa rats fed Baked Salmon Diet when compared to those fed the Control Diet may be a consequence of the slow clearance of chylomicrons in this rat model [24,25], as the fatty acid content in serum triacylglycerols mirrors the dietary fatty acids to a great extent despite a 12 h fasting period before blood samples were collected. However, since the serum apoB-48 concentration was similar in Zucker fa/fa rats fed Baked Salmon Diet or Control Diet, this suggest that the number of chylomicrons may be similar in the two groups but the size of chylomicrons may be bigger in rats fed Baked Salmon Diet, i.e. with a higher ratio of triacylglycerols to apoB-48. The serum apoB-100 concentration was actually lower in Zucker fa/fa rats fed the Baked Salmon Diet when compared to those fed Control Diet, possibly a consequence of reduced secretion of VLDL from the liver caused by fish oil in the Baked Salmon Diet, as n−3 PUFA has been shown to hamper VLDL-secretion [38]. ApoB-100 is an important apolipoprotein in both VLDL and LDL, and the lower serum total cholesterol and LDL cholesterol concentrations in Zucker fa/fa rats fed Baked Salmon Diet is thus in line with lower serum apoB-100 concentration. It is well known that insulin inhibits the secretion of apoB-100 from rat hepatocytes and stimulate its degradation [41], however no differences were seen between serum fasting concentrations of insulin in Zucker fa/fa rats fed Baked Salmon Diet or Control Diet, further supporting the assumption that higher triacylglycerol in Zucker fa/fa rats fed Baked Salmon Diet is caused by a combination of higher fat intake from salmon filet and slower clearance of chylomicrons and was not a consequence of increased VLDL secretion. Contrary to this, no differences were seen between Long-Evans rats fed the Baked Salmon Diet and the Control Diet regarding serum concentrations of triacylglycerols, cholesterol, apoB-48 and apoB-100, thus suggesting that the Baked Salmon Diet did not affect VLDL-secretion or degradation of lipoproteins including chylomicrons in rats with normal lipid metabolism.

This study has some limitation. We wanted the Long-Evans rats in this follow-up study to reach the same bodyweight as the Zucker rats after four weeks intervention, which was 554 ± 26 g, and we achieved a mean body weight for all Long-Evans rats at endpoint of 572 ± 38 g, with no difference between the experimental groups (*p* ANOVA = 0.062). Since Long-Evans rats gain weight more slowly compared to Zucker fa/fa rats, the Long-Evans rats were older compared to Zucker fa/fa at the end of the intervention period (17–19 weeks old vs. 12–13 weeks old). Another limitation is that, since the Long-Evans experiment was conducted after the Zucker fa/fa study, the experimental conditions may have been different, although we made great efforts to avoid such biases by controlling temperature, humidity, using the same cages, using the same frozen feeds, and the same staff members handled and euthanized the rats.

## 5. Conclusions

Our findings show that a diet containing baked salmon fillet had different effects on serum triacylglycerol and cholesterol concentrations and fatty acid composition in the hyperlipidemic obese Zucker fa/fa rat and in the normolipidemic Long-Evans rat, despite similar energy intake and bodyweight in these two rat strains at endpoint. Zucker fa/fa rats fed the Baked Salmon Diet had higher serum triacylglycerol concentration and lower serum cholesterol when compared to Zucker fa/fa rats fed the Control Diet, whereas no differences were seen in serum triacylglycerol and cholesterol concentrations between Long-Evans rats fed these two diets. Based on the findings that fatty acids esterified as triacylglycerols had great similarities to the composition of dietary fatty acids and the similar concentration of apoB-100 between the experimental groups in the Zucker fa/fa experiment, the higher serum triacylglycerol concentration in Zucker fa/fa rats fed Baked Salmon Diet may be a consequence of slow clearance of chylomicrons in this rat strain combined with more fat originating from salmon fillet in the Baked Salmon Diet. The lower serum cholesterol concentration combined with lower apoB-100 concentration suggest lower VLDL-secretion in Zucker fa/fa rats fed Baked Salmon Diet, and therefore higher triacylglycerol concentration in these rats is probably not caused by more VLDL in circulation.

## Figures and Tables

**Table 1:** Composition of the experimental diets.

g/kg Diet	Control Diet	Baked Salmon Diet
Casein protein ^1^	216.2	162.2
Freeze dried baked salmon ^2^	-	100.0
Cornstarch	512.0	466.0
Sucrose	90.0	90.0
Cellulose	50.0	50.0
Soybean Oil	70.0	70.0
t-Butylhydroquinone	0.014	0.014
Mineral Mix (AIN-93-MX)	35.0	35.0
Vitamin Mix (AIN-93-VX)	10.0	10.0
L-Methionine	1.60	1.60
L-Cystine	3.0	3.0
Choline Bitartrate ^3^	2.5	2.5
Growth and Maintenance Supplement ^4^	10.0	10.0

^1^ Contains 92.5% crude protein; ^2^ contains 50% crude protein; ^3^ contains 41% choline; ^4^ contains vitamin B12 (40 mg/kg) and vitamin K1 (25 mg/kg) mixed with sucrose (995 g/kg) and dextrose (5 g/kg).

**Table 2:** Fatty acid contents in the experimental diets ^1^.

g/kg Diet	Control Diet	Baked Salmon Diet
16:0	7.5	9.6
18:0	2.5	3.0
18:1n−7	0.87	1.6
18:1n−9	13.9	24.8
18:2n−6	32.7	34.5
18:3n−3	3.9	5.0
20:5n−3	ND	0.53
22:5n−3	ND	0.22
22:6n−3	ND	1.0

^1^ Showing fatty acids present in amounts >0.2 g/kg, ND; not detected.

**Table 3:** Relative weight of WAT, energy intake, and fat intake is a table.

	Zucker fa/fa Rats			Long-Evans Rats		
	Control Group	Baked Salmon Group	*p* Diets	Control Group	Baked Salmon Group	*p* Diets
Relative amount of WAT ^1^, g/100 g bodyweight	7.43 ± 0.75	7.58 ± 0.80	0.77	4.97 ± 0.90	5.80 ± 1.32	0.23
Energy intake, kcal/24 h	144 ± 17	126 ± 20	0.15	123 ± 18	122 ± 21	0.91
Fat intake, g/24 h	2.7 ± 0.3	3.3 ± 0.5	0.045	2.3 ± 0.3	3.2 ± 0.5	0.0062

Values are mean and standard deviations, *n* = 5 in Zucker fa/fa Control Group, and *n* = 6 in all other groups. *p* < 0.05 was considered significant. Groups within rat strains are compared using Independent Samples T Test assuming equal variances. ^1^ The sum of epididymal, renal and retroperitoneal white adipose tissue from both sides of the rat, relative to bodyweight.

**Table 4:** Serum concentrations of lipids, total bile acids, apolipoproteins B48 and B100, and CRP.

	Zucker Fa/Fa Rats			Long-Evans Rats		
	Control Group	Baked Salmon Group	*p* Diets	Control Group	Baked Salmon Group	*p* Diets
Total cholesterol, mmol/L	6.3 ± 0.6	4.9 ± 0.8	0.011	2.9 ± 0.3	2.8 ± 0.5	0.58
HDL cholesterol, mmol/L	5.3 ± 0.3	2.7 ± 0.2	4.4 × 10^−8^	2.5 ± 0.2	2.3 ± 0.2	0.18
LDL cholesterol, mmol/L	1.5 ± 0.3	0.9 ± 0.3	0.0025	0.7 ± 0.1	0.6 ± 0.1	0.21
Cholesteryl ester, mmol/L	4.9 ± 0.5	3.7 ± 0.5	0.0045	2.2 ± 0.2	2.2 ± 0.4	0.74
Total bile acids, umol/L	46 ± 21	16 ± 7	0.0084	7 ± 5	5 ± 4	0.44
NEFA, mmol/L	0.91 ± 0.13	0.84 ± 0.12	0.38	0.43 ± 0.18	0.19 ± 0.14	0.030
ApoB-100, ng/mL	163 ± 53	96 ± 32	0.036	61 ± 6	68 ± 11	0.22
ApoB-48, ng/mL	7 ± 3	10 ± 3	0.12	20 ± 3	21 ± 4	0.69
CRP, mg/mL	1.18 ± 0.08	0.96 ± 0.14	0.014	0.96 ± 0.05	0.98 ± 0.07	0.62

Values are mean and standard deviations, *n* = 5 in Zucker fa/fa Control Group, and *n* = 6 in all other groups. *p* < 0.05 was considered significant. Groups within rat strains are compared using Independent Samples T Test assuming equal variances. HDL, high-density lipoprotein; LDL, low-density lipoprotein; NEFA, non-esterified fatty acids; Apo, apolipoprotein; CRP, C-reactive protein.

## Supplementary Materials

The following is available online at http://www.mdpi.com/2072-6643/10/10/1459/s1, Table S1: Selected fatty acids esterified as triacylglycerols and phospholipids.
