# Supplementary material for: Salmon Fillet Intake Led to Higher Serum Triacylglycerol in Obese Zucker Fa/Fa Rats But Not in Normolipidemic Long-Evans Rats
Source: Nutrients. 2018 Oct 8;10(10):1459. doi: 10.3390/nu10101459 (PMC6213735; doi:10.3390/nu10101459)
Supplement: Supplementary file 1 [file nutrients-10-01459-s001.pdf]

Supplemental Table 1: Selected fatty acids[1] esterified as triacylglycerols and phospholipids

g/100g fatty acids	Zucker fa/fa rats Control Group	Baked Salmon Group	P diets	Long-Evans rats Control group	Baked Salmon group	P diets
Triacylglycerols						
16:0	32.9 ±3.2	32.4 ±1.6	0.76	27.7 ±1.9	26.9 ±3.5	0.63
18:0	3.12 ±0.39	2.77 ±0.46	0.21	4.95 ±1.86	4.76 ±2.44	0.88
ΣSFA[2]	38.3 ±3.2	37.1 ±2.0	0.47	34.9 ±3.9	33.8 ±5.1	0.69
16:1n-7	8.82 ±1.25	7.19 ±0.75	0.88	5.21 ±0.95	4.32 ±1.83	0.31
16:1n-9	0.76 ±0.10	0.76 ±0.07	0.88	0.57 ±0.08	0.49 ±0.05	0.087
18:1n-7	4.21 ±0.37	3.48 ±0.28	$4.7x10^{-3}$	3.18 ±0.48	2.73 ±0.46	0.13
18:1n-9	27.3 ±1.4	26.8 ±1.1	0.50	22.7 ±2.2	22.8 ±3.6	0.94
ΣMUFA[3]	41.7 ±1.8	38.9 ±1.8	0.026	32.0 ±2.8	30.9 ±5.2	0.66
18:3n-3	1.00 ±0.41	1.17 ±0.22	0.42	1.51 ±0.23	1.73 ±0.40	0.26
20:5n-3	0.44 ±0.12	1.32 ±0.35	$4.2x10^{-4}$	0.69 ±0.22	2.02 ±0.92	$6.5x10^{-3}$
22:5n-3	0.78 ±0.08	1.78 ±0.36	$1.8x10^{-4}$	0.56 ±0.17	1.21 ±0.52	$4.7\ x10^{-3}$
22:6n-3	1.09 ±0.16	3.87 ±0.83	$4.2x10^{-5}$	1.45 ±0.41	3.47 ±1.42	$7.5\ x10^{-3}$
Σn-3 PUFA[4]	3.3 ±0.6	8.2 ±1.7	$1.6x10^{-4}$	4.2 ±0.9	8.6 ±3.1	0.0072
18:2n-6	12.3 ±2.9	12.29 ±1.29	0.99	22.8 ±2.9	21.9 ±3.2	0.63
20:3n-6	0.38 ±0.03	0.59 ±0.08	$2.9x10^{-4}$	0.23 ±0.13	0.33 ±0.20	0.33
20:4n-6	2.37 ±0.21	1.79 ±0.51	0.042	4.61 ±0.55	3.39 ±0.73	$8.5x10^{-3}$
22:4n-6	0.69 ±0.12	0.48 ±0.13	0.019	0.46 ±0.15	0.37 ±0.08	0.23
Σn-6 PUFA[5]	16.6 ±2.8	15.9 ±1.8	0.60	29.0 ±3.5	26.7 ±4.0	0.33
Phospholipids						
16:0	18.1 ±1.1	17.1 ±1.4	0.26	22.4 ±1.3	21.5 ±2.5	0.49
18:0	27.0 ±1.2	29.2 ±1.8	0.048	23.6 ±1.00	25.8 ±1.8	0.034
24:0	0.93 ±0.09	0.56 ±0.08	$3.8x10^{-5}$	1.26 ±0.19	0.96 ±0.16	0.013
ΣSFA[2]	47.5 ±1.0	48.1 ±0.5	0.25	49.4 ±1.0	50.1 ±1.2	0.25
16:1n-7	0.83 ±0.15	0.63 ±0.11	0.030	0.43 ±0.043	0.31 ±0.06	$2.6\ x10^{-3}$
18:1n-9	3.72 ±0.55	3.12 ±0.63	0.13	2.75 ±0.24	2.64 ±0.12	0.34
18:1n-7	2.45 ±0.25	1.82 ±0.30	$4.7x10^{-3}$	2.33 ±0.17	1.66 ±0.29	$6.5x10^{-4}$
24:1n-9	0.94 ±0.10	1.12 ±0.17	0.069	0.95 ±0.16	1.53 ±0.25	$6.9x10^{-4}$
ΣMUFA[3]	8.2 ±0.8	7.0 ±1.0	0.059	6.7 ±0.3	6.5 ±0.4	0.24
20:5n-3	0.11 ±0.02	0.49 ±0.12	$7.0x10^{-5}$	0.09 ±0.02	0.35 ±0.13	$6.2x10^{-4}$
22:5n-3	0.70 ±0.06	0.74 ±0.06	0.31	0.49 ±0.06	0.67 ±0.08	$1.4\ x10^{-3}$
22:6n-3	4.53 ±0.22	6.73 ±0.43	$3x10^{-6}$	4.81 ±0.39	5.91 ±0.69	$6.8\ x10^{-3}$
Σn-3 PUFA[4]	5.4 ±0.2	8.0 ±0.4	$3.4x10^{-7}$	5.4 ±0.4	7.0 ±0.8	$1.3x10^{-3}$
18:2n-6	8.00 ±0.36	9.51 ±1.94	0.12	7.92 ±0.77	8.38 ±0.34	0.21
20:3n-6	1.05 ±0.16	2.10 ±0.48	$1.3x10^{-3}$	0.55 ±0.10	0.97 ±0.32	0.013
20:4n-6	28.2 ±0.6	24.6 ±2.6	0.014	28.9 ±1.2	26.3 ±0.9	$1.6\ x10^{-3}$
22:5n-6	0.79 ±0.13	0.19 ±0.04	$2.4x10^{-6}$	0.22 ±0.08	0.08 ±0.01	$2.5\ x10^{-3}$
Σn-6 PUFA[5]	38.9 ±0.4	37.0 ±0.7	$6.4x10^{-4}$	38.5 ±0.6	36.4 ±1.1	$2.4x10^{-3}$

[1]Showing fatty acids present in amounts >0.3 g/100g fatty acids in at least one of the experimental groups.

[2]Sum of 12:0, 14:0, 15:0, 16:0, 17:0, 18:0, 20:0, 22:0, 23:0, 24:0.

[3]Sum of 14:1n-5, 16:1n-7, 16:1n-9, 17:1n-8, 18:1n-5, 18:1n-7, 18:1n-9, 20:1n-9, 24:1n-9.

[4]Sum of 18:3n-3, 20:3n-3, 20:5n-3, 22:5n-3, 22:6n-3.

[5]Sum of 18:2n-6, 18:3n-6, 20:3n-6, 20:4n-6, 22:4n-6, 22:5n-6.

Values are mean and standard deviations, n= 5 in Zucker fa/fa Control group, and n=6 in all other groups.

$p < 0.05$ were considered significant. Groups within rat strains are compared using Independent Samples T Test assuming equal variances.
